# A dual-flow RootChip enables quantification of bi-directional calcium signaling in primary roots

**DOI:** 10.3389/fpls.2022.1040117

**Published:** 2023-01-10

**Authors:** Claudia Allan, Ayelen Tayagui, Rainer Hornung, Volker Nock, Claudia-Nicole Meisrimler

**Affiliations:** ^1^ School of Biological Sciences, University of Canterbury, Christchurch, New Zealand; ^2^ Department of Electrical and Computer Engineering, University of Canterbury, Christchurch, New Zealand; ^3^ MacDiarmid Institute for Advanced Materials and Nanotechnology, Wellington, New Zealand; ^4^ imec Netherlands, Holst Centre, Eindhoven, Netherlands

**Keywords:** abiotic stress, calcium, signalling, *Arabidopsis*, microfluidics, root, osmotic stress

## Abstract

**One sentence summary:** Bi-directional-dual-flow-RootChip to track calcium signatures in *Arabidopsis* primary roots responding to osmotic stress.

Plant growth and survival is fundamentally linked with the ability to detect and respond to abiotic and biotic factors. Cytosolic free calcium (Ca^2+^) is a key messenger in signal transduction pathways associated with a variety of stresses, including mechanical, osmotic stress and the plants’ innate immune system. These stresses trigger an increase in cytosolic Ca^2+^ and thus initiate a signal transduction cascade, contributing to plant stress adaptation. Here we combine fluorescent G-CaMP3 *Arabidopsis thaliana* sensor lines to visualise Ca^2+^ signals in the primary root of 9-day old plants with an optimised dual-flow RootChip (dfRC). The enhanced polydimethylsiloxane (PDMS) bi-directional-dual-flow-RootChip (bi-dfRC) reported here adds two adjacent inlet channels at the base of the observation chamber, allowing independent or asymmetric chemical stimulation at either the root differentiation zone or tip. Observations confirm distinct early spatio-temporal patterns of salinity (sodium chloride, NaCl) and drought (polyethylene glycol, PEG)-induced Ca^2+^ signals throughout different cell types dependent on the first contact site. Furthermore, we show that the primary signal always dissociates away from initially stimulated cells. The observed early signaling events induced by NaCl and PEG are surprisingly complex and differ from long-term changes in cytosolic Ca^2+^ reported in roots. Bi-dfRC microfluidic devices will provide a novel approach to challenge plant roots with different conditions simultaneously, while observing bi-directionality of signals. Future applications include combining the bi-dfRC with H_2_O_2_ and redox sensor lines to test root systemic signaling responses to biotic and abiotic factors.

## 1 Introduction

Physiological processes in eukaryotic organisms rely on a functional signal transduction system to coordinate external and internal signals resulting in appropriated response. External signals, including abiotic and biotic stress, depend on initial perception by cell-surface receptors, cellular transmission and translation that allow plants to balance external fluctuations in an ever-changing environment. Calcium (Ca^2+^) is an essential macronutrient in plants, where it is a component of cell walls, membranes, proteins and finally, yet importantly, a key messenger involved in signal transduction (Bergey et al., 2014). Changes in cytosolic free Ca ion ([Ca^2+^]cyt) concentrations serve as a signal, which translates into downstream responses (McAinsh and Pittman, 2009; Dodd et al., 2010; Thor and Peiter, 2014). These signals are known to be associated with different patterns of transient, sustained, or oscillatory rises in [Ca^2+^]cyt – comparable to a Morse code (De Koninck and Schulman, 1998; McAinsh & Pittman, 2009). Commonly, a message is converted into a Ca^2+^ signal (transmissible form), followed by diverse transmission and transduction of the signal, which associates with a decoding machinery allowing the cell to interpret Ca^2+^ signatures generated under different environmental stresses (Allan et al., 2022a). The coding function relies on maintaining constantly low levels of [Ca^2+^]cyt around 0.1 μM. Buffers, H^+^/Ca^2+^ antiporters and Ca^2+^-ATPases, actively remove Ca^2+^ from the cytosol into the apoplast or intracellular stores (Connorton et al., 2012; Bonza et al., 2016). In tandem, Ca^2+^ can move down the concentration gradient into the cytosol through channel proteins, subsequently generating a signal. In order to code for downstream pathways, like gene (de-) activation, the signal must be interpreted and communicated into a cellular response *via* calcium binding proteins (CBPs), e.g. Ca^2+^-Dependent Protein Kinases (CPKs) and Calcineurin-B Like proteins (CBLs) (Bergey et al., 2014; Wagner et al., 2015; Thoday-Kennedy et al., 2015; Zhang et al., 2016; Zhu et al., 2016).

So far, the majority of [Ca^2+^]cyt measurements and experiments have been accomplished in *Arabidopsis thaliana (Arabidopsis)*. In recent years a variety of approaches have been used for this, including the luminescent Ca^2+^-interacting aequorin protein, (Knight et al., 1991), G-CaMP3 (Toyota et al., 2018), as well as ratiometric biosensors like the MatryoshCaMP6s calcium sensors (Ast et al., 2017). The visualisation of cytosolic calcium transients using luminescent or fluorescent sensors has revealed stimulus‐specific calcium signatures and long‐distance communication in roots (Knight et al., 1997; Kiegle et al., 2000; Krebs et al., 2012; Choi et al., 2014; Xiong et al., 2014; Behera et al., 2015; Keinath et al., 2015). However, mimicking complex soil environments that roots are exposed to, whilst being able to control these conditions and quantify the effects, has been technically challenging. To overcome these challenges, microfluidic approaches have been combined with advanced microscopy in recent years. The use of microfluidics has been shown to allow for dynamic experiments that mimic environmental complexity found in the soil in a high spatio-temporal resolution on organismal and cellular level for force sensing and flow stream shaping of tip growing organisms (Bhatia & Ingber, 2014; Nezhad, 2014; Zheng et al., 2016; Tayagui et al., 2017; Sun et al., 2020). Particularly significant breakthroughs have been made by the introduction of ‘soil‐on‐a‐chip’ microfluidic technologies to investigate root-microbe interactions and the dual-flow-RootChip (dfRC) platform, the latter which allows for the cultivation of *Arabidopsis* roots in asymmetric microenvironments (Stanley and van der Heijden, 2017; Stanley et al., 2018). Building on this platform, we have previously reported the basic chip design (Allan et al., 2022b) and operation of a bi-directional-dual-flow-RootChip (bi-dfRC). In the following we expand on the platform and demonstrate the use of the bi-dfRC to study the directionality and role of Ca^2+^ signaling in plant roots in response to stress factors. The bi-dfRC expands on conventional dfRCs (Stanley et al., 2018) by adding bi-directional flow capabilities for guided root growth and controlled exposure of the root to independent or asymmetric solute gradients. To probe for Ca^2+^ directional localisation in *Arabidopsis* root systems, a G-CaMP3 line (Toyota et al., 2018) was combined with the polydimethylsiloxane (PDMS) root chip technology. We demonstrate that a bi-directional flow system allows for studying directionality of Ca^2+^ signals upon selective application of stresses from adjacent directions. These findings are discussed in context with existing literature on varying Ca^2+^ sensing machinery in plant roots that may allow to distinguish local stress application and elicit downstream long-distance systemic signaling as a response, thus fine-tuning adaptation processes.

## 2 Methods

### 2.1 Bi-directional-dual-flow-RootChip fabrication

Microfluidic chips were fabricated using photolithography and replica molding (Orcheston-Findlay et al., 2018). In brief, designs were created using software (Mentor Graphics, v2020.1), and transferred to photo-masks (Nanofilm) using a laser mask writer (Heidelberg μPG101) and wet-etching. Single-side polished silicon wafers (4”, Prime grade, WaferPro) were dehydrated in an oven (Hearatherm) at 180°C for 24 hours and cleaned in a O_2_-plasma cleaner (PIE Scientific Tergeo) at 100 W for 10 min. After cleaning, dry-film, negative-tone photoresist (SUEX 100, DJMicrolaminates) was laminated (Sky-335R6) onto the wafer. The chip design was then transferred into the photoresist by exposure of the prepared photomask in a mask aligner (MA-6, SUSS MicroTec). After exposure to UV light, the wafer was baked on a hot plate (HS40, Torrey Pines Scientific) for 5 min at 65°C and 20 min at 95°C. Transferred chip structures were developed in propylene glycol methyl ether acetate (PGMEA) developer for 30 min and rinsed with isopropanol for 5 min. Lastly, the wafer was hard-baked on the hot plate for 1 hour at 125°C, yielding a mold ready for replica-molding (Xia and Whitesides, 1998).

Prior to molding, the silicon wafer was pre-treated with an anti-adhesion agent (Trichloro (1H,1H,2H,2H-perfluorooctyl) silane, Sigma-Aldrich) to prevent sticking of the elastomer cast during the subsequent replica-molding. This step was repeated after 10 subsequent uses of the wafer as mold master. For replica-molding, PDMS (Sylgard 184, Electropar) pre-polymer silicone elastomer base was mixed thoroughly with silicone elastomer curing agent at a 10:1 (w/w) ratio and degassed to remove air bubbles. Once cast onto the wafer mold, the PDMS was degassed again and placed on a hot plate at 80°C for 2 hours to cure. Subsequently, the PDMS was carefully peeled off from the wafer and cured on the hot plate for a further 2 hours at 80°C. Following the second curing bake, inlet and outlet holes were core-punched (Ø1 mm & 3 mm, ProSciTech) and the PDMS was cut into individual bi-dfRC chips using a guillotine or scalpel. Chips were then O_2_-plasma activated (PIE Scientific Tergeo) at 15 W power for 1 minute and bonded to pre-cleaned microscope slides (26×60 mm, Lab Supply) by lightly pressing the exposed surfaces of the PDMS and glass together. Bonded chips were then baked for 2 hours on a hot plate at 80°C to strengthen the bond.

To permanently retain the hydrophilicity of microfluidic channels (Hemmilä et al., 2012; Hashemi et al., 2017) and reduce diffusion of small molecules into PDMS, assembled chips were placed in the plasma cleaner (PIE Scientific Tergeo) and exposed to 30 W power for 3 min. Following activation, 22% w/v polyvinylpyrrolidone (PVP, Sigma-Aldrich) in DI water was pipetted into the microchannels for 1 minute to permanently alter the hydrophilic retention of PDMS. Microchannels were then washed with DI water and dried using a pressurised nitrogen gun. For storage, chips were placed in a vacuum desiccator for 3 hours, then sealed shut using vacuum-sealable food storage bags (Sunbeam). In order to obtain high-quality images of the microfluidic device microchannels an epoxy dye protocol was used (Soffe et al., 2020). For this, Sudan dye (Sigma-Aldrich) was added to 1 mL of toluene (Sigma-Aldrich) and Norland Optical Adhesive (NOA72, Norland Products). After toluene evaporated, 60 µL of the dye was added to the inlet and outlet channels of the bi-dfRC using a pipette. The dye entered the inlet channels *via* passive pumping (Hosokawa et al., 2004) and was cured using a spot UV curing system (OmniCure^®^ S2000).

### 2.2 Asymmetric co-flowing solutions

A dual-pressure syringe pump system (NE-1010, New Era Pump Systems Inc) was used to deliver singular or dual treatment into the bi-dfRC observation chamber (OC). The pump system holds syringes (BD, MediRay) connected to the chip via 1/16” OD ethylene tetrafluoroethylene (ETFE) tubing (Kinesis), primed with a silicone tubing sleeve (Darwin Microfluidics) and short 1.5 mm OD metal tubes. Flow rate was set to 20 μL per minute.

### 2.3 Seed vernalisation and germination on the bi-directional-dual-flow-RootChip

In this study, Col-0 and G-CaMP3 *Arabidopsis* lines (originating from Toyota et al., 2018) were treated similarly throughout all experiments. *S*eeds were sterilised in 0.1% Triton X-100 for 3 min, followed by 70% ethanol (EtOH) for 2 min, subsequently washed 4 times with sterile Milli-Q^®^ water (Merck). Pre-sterilised seeds were vernalised in water at 4°C for approximately 12 hours. Seeds were cultured onto half-strength Murashige and Skoog (½ MS) medium (Duchefa), 0.31 mM MES (Sigma-Aldrich) and 8% agarose. Plants on plates were grown for 4-days at a short-day cycle (8 h light, 16 h dark) with 65% humidity and 150 µmol m^-2^ s^-1^ per µA light intensity. On chip sub-culturing was achieved by transferring 4-day old *Arabidopsis* plants directly onto the pre-sterilised bi-dfRCs. Prior to sub-culturing, microchannels and inlets were pre-sterilised with 70% ethanol, and then washed with ½ MS/0.31 mM MES liquid medium. Agarose squares (4×4 mm) were oriented above the root inlet channel. Plants were aligned with the agarose squares and primary roots were carefully situated into the root inlet channel. Chips were stored in plastic incubation chambers (Nunc OmniTray single-well plates; Thermo Fisher Scientific), surrounded by sterile Milli-Q water, for humidity. The set-up was incubated for 5-days under the pre-alluded short-day cycle (8 h light; 16 h dark). The set up was tilted at a 45-degree angle to promote root growth into the bi-dfRC microchannel.

### 2.4 Fluorescence microscopy, image editing and quantification analysis

For epifluorescence microscopy, all images and videos were obtained with a Zeiss (AX10) 5x lens (EC Plan-Neofluar 5x/0.15 M27) (**Supplemental Figure 1A**). Ca^2+^ fluorescence was observed with an eGFP filter (38 HE Green Fluorescent Prot BP 450-590) and bright field (BF) was set to 2.7 ms exposure, TL lamp 30%. Images and videos were analysed either in in Fiji-ImageJ (Schindelin et al., 2012) or ZEN Blue software (Zeiss). *Arabidopsis* root tip quantification was accomplished utilising the freehand selection tool. Treatments were applied as either a control solution (½ Murashige and Skoog (MS) medium, plus 0.31 mM 2-(N-morpholino) ethanesulfonic acid (MES) dissolved in Milli-Q^®^ water) or stress solution (100 mM sodium chloride (NaCl) or 20% polyethylene glycol dissolved in ½ MS/0.31mM MES media). For raw data collection fluorescence intensity was measured in the original raw video frames. Linear section 1 was sampled at the root tip columella cells, while linear sections 2 and 3 represent meristematic and elongation zone 1 and 2(ME1 and ME2) of the root, respectively, and linear sections 4 and 5 represent elongation and differentiation zone 1 and 2 (ED1 and ED2) of the root, respectively (**Supplemental Figure 1B**). Linear quantification was carried out with the straight-segmented tool. Five linear sections were aligned adjacently per sample, with an intersection space of 290 µm between sites 1 to 4, and 580 µm between site 4 and 5 (**Supplemental Figure 1B**). An analogue-to-digital conversion in ImageJ (Fiji) was used to convert evenly spaced values into a signal. Additionally, the ROI Manager, Multi Measure tool was used for a rapid semi-automatic measurement of all frames in the video. Fluorescent plot profiles at each linear section and time interval were analysed into raw data files in Excel (16.61.1 (22052000), Microsoft). Statistical analysis and graphs were conducted in Prism (V8.4.3, GraphPad). Original videos have been compressed to reduce size below 30 MB/video. Videos were exported as AVI M-JPEG compression with 100% quality and set to 30 fps, in Zen blue. Original videos are 568 images taken over 3 minutes and 3.3 fps.

### 2.5 Kymograph generation

Kymographs were used to show spatial intensity over time for exemplified roots. For this, original.czi files were converted into.avg video formats using Zen blue software, without compression. Every picture frame was defined as a time-point. The fluorescence intensity (corresponds to cytosolic Ca^2+^) of transverse pixel sections was summed up for each spatio-temporal position.

An in-house python script was developed with PyCharm (www.jetbrains.com) in Python ver. 3.6 (https://www.python.org/downloads/release/python-360/) to generate kymographs. The main functionalities were provided by the open-source computer vision library OpenCV (https://pypi.org/project/opencv-python/). The script reads out every picture within the video and applies a summation longitudinal or across the root in a manually predefining region of interest (ROI). Operation of the script is controlled *via* command line interface using the standard library argparse (https://docs.python.org/3/library/argparse.html).

## 3 Results and discussion

Site-specific exposure of environmental stress to plant roots has the potential to yield new insight into how plants detect and respond to abiotic and biotic factors. To study the role of Ca^2+^ signals, existing dfRC technology was adapted and extended in this research to enable bi-directional stimulation. The enhanced bi-dfRC allows for the cultivation of *Arabidopsis* roots to capture root elongation, cellular localisation and dispersion patterns of fluorescent signals of any kind. We used G-CaMP3 expressing *Arabidopsis* plants to observe NaCl and PEG-induced Ca^2+^ signals in the primary root. The need to modify a bi-dfRC design became apparent during initial experiments related to Ca^2+^ quantification with the conventional dfRC based on the design by Stanley et al., 2018 (data not shown). During these early NaCl treatment experiments, it became evident that a chip, which also enables probing from the root tip, would be of significant advantage. Such a device would allow users to quantify the signal in a spatio-temporal manner, in dependence on the first contact site, as well as with different solutions on each side of a root. This is of importance when elucidating difference in signal dispersion within the system, which can then be used as the basis for modelling approaches. By utilising G-CaMP3 lines for this, results can be compared to prior observations found with varying intensiometric calcium indicators, including R-GECO1 lines (Stanley et al., 2018). In particular, two different osmolytes, NaCl and PEG, were used in comparison to control treatment to explore Ca^2+^ responses in roots within 180 s post-treatment application.

### 3.1 Bi-directional-dual-flow-RootChip design

**Figure 1:**
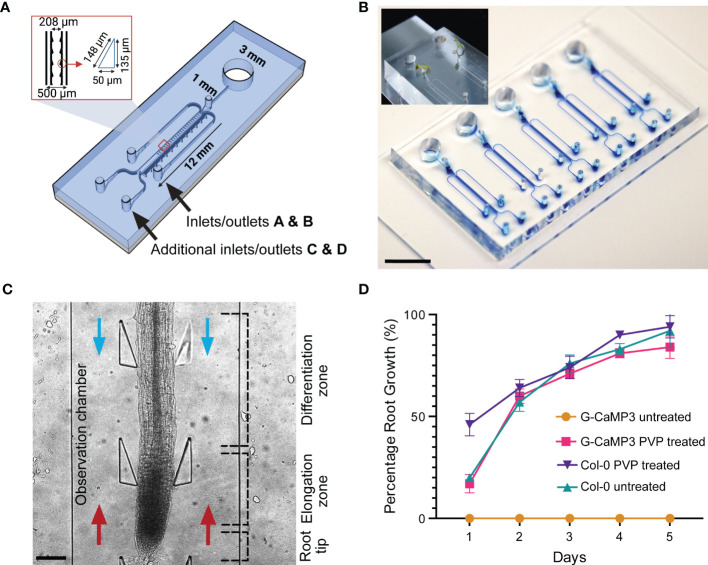
Bi-directional dual-flow-RootChip (Bi-dfRC) for laminar flow perfusion of stress treatments at the tip and differentiation zone of *Arabidopsis* roots. **(A)** Schematic diagram of the bi-dfRC including pillar array dimensions (identical to the conventional dfRC) in addition to a second set of inlets/outlets C & D for bi-directional stress application at the tip or differentiation zone. **(B)** Image of epoxy dye (blue) filled microchannels for visualisation of root observation chamber joining 4 inlet/outlets. 9-day old *Arabidopsis* G-CaMP3 plants cultured into the root inlet of the bi-dfRC depicted top left (scale bar 10 mm). **(C)** *Arabidopsis* G-CaMP3 root situated in the bi-dfRC observation chamber highlighting key zones (tip, elongation, and differentiation zone) and treatment orientation (blue arrows from top inlets A & B, red arrows from bottom inlets C & D, scale bar 200 µm). **(D)** Average percentage of *Arabidopsis* G-CaMP3 and Col-0 root growth into PVP-treated and untreated microchannels over 5-days (n= 100).

Identical in dimension to the conventional dfRC design (Stanley et al., 2018), the bi-dfRC adds a second adjacent set of externally accessible microchannels (Allan et al., 2022b), which are connected to the base of the root observation chamber (OC) for flow reversal (**Figure 1A**). As shown in **Figure 1A**, the root OC itself contains 34 pairs of triangular elastomeric micropillars for root guidance and force sensing. Connected to each OC are a singular media port and plant seeding area for root growth (**Figure 1B**), as well as the four media inlets/outlets (A & B top, C & D bottom) for chemical treatment application. As indicated in **Figure 1C**, using these extra inlets/outlets, chemical treatment can either be applied through inlets A and B for application of solutions from the differentiation (shoot) site or through inlets C and D for application of solution from the root tip (root) site. In either case, the other inlets then become the outlets, thus adding bi-directional treatment capabilities for targeted application of stress conditions in varying local root tissues. Independent bi-dfRCs were combined into one PDMS device to yield one to five parallel OCs on a single glass substrate (**Figure 1B**). Following sub-culture of 4-day old *Arabidopsis* roots from plant culture into the bi-dfRC, the roots grow into the OCs over another 5-days (**Figure 1B**), upon which stress treatments can be applied at the differentiation zone or tip (**Figure 1C**).

### 3.2 Hydrophilic retention of microchannels

Many PDMS microfluidic systems require hydrophilic retention or water retention to promote protrusion of tip growing organisms into fluidic microchannels (Halldorsson et al., 2015). PDMS chips however, naturally exhibit hydrophobic or water repelling surface properties in their native form (Hemmilä et al., 2012). This is in contrast to the glass base of the bi-dfRC, which typically has hydrophilic surface properties. By combining the two materials during chip fabrication, the resulting devices and OCs present a partially hydrophobic environment without further treatment (Guckenberger et al., 2014). We observed that devices with exposed hydrophobic PDMS surfaces directly hindered the root growth of the G-CaMP3 sensor line into bi-dfRC devices. Interestingly, the same surface properties had no impact on the growth of wild type Col-0 roots in our work (**Figure 1D**) and no growth inhibition for the R-GECO1-expressing *Arabidopsis* roots has been reported (Stanley et al., 2018). This difference in behavior may relate to sensing mechanisms of hydrophobic, hydrophilic, ionic and non-ionic surfaces by roots. The fully impaired protrusion of roots from G-CaMP3 lines into partially hydrophobic and hydrophilic surfaces points to a disturbance of surface sensing and charge in these lines, and the involvement of Ca^2+^ in this process. A known property of G-CaMP3 sensor lines is enhanced kinetics for high-speed Ca^2+^ imaging (Russell, 2011). Hence, G-CaMP3 may quench free cytosolic Ca^2+^ away from other essential physiological processes including stomatal closure, water-sensing mechanisms and sensing of varying surfaces (Cho et al., 2017). It has been reported that hydrophobic soil increases with lower pH, whereas hydrophilic soils are retained at a pH of approximately 6.5 and higher (Vogelmann et al., 2013). While, together with our observation of growth inhibition, this suggests that a fully hydrophilic microchannel should re-initiate G-CaMP3 root growth, further insight is needed to understand the details of how roots sense surface charge, hydrophobicity and other properties, as well as the role of Ca^2+^ in these important processes.

To test whether modified surface properties would facilitate G-CaMP3 root growth, partially hydrophobic microchannels were initially made temporarily hydrophilic *via* plasma activation, a method which exposes given surfaces to oxygen plasma (Jokinen et al., 2012). However, PDMS only retains this plasma-activated surface modification for minutes to hours, which severely limits its use for long-term culture (Plegue et al., 2018). To permanently preserve the hydrophilic surface, PVP - a polymer that reduces the hydrophobicity of surface particles (Kao et al., 2003), was reacted with the plasma-activated PDMS (Hashemi et al., 2017). This rendered the PDMS, and thus the bi-dfRC device OCs, permanently hydrophilic. As is demonstrated by **Figure 1D**, plasma-activated but PVP untreated G-CaMP3 root growth did not grow into the bi-dfRC OCs. In contrast, devices treated with PVP did not exhibit any growth limitations on G-CaMP3 roots.

### 3.3 Mechanics underpinning fluid flow control

**Figure 2:**
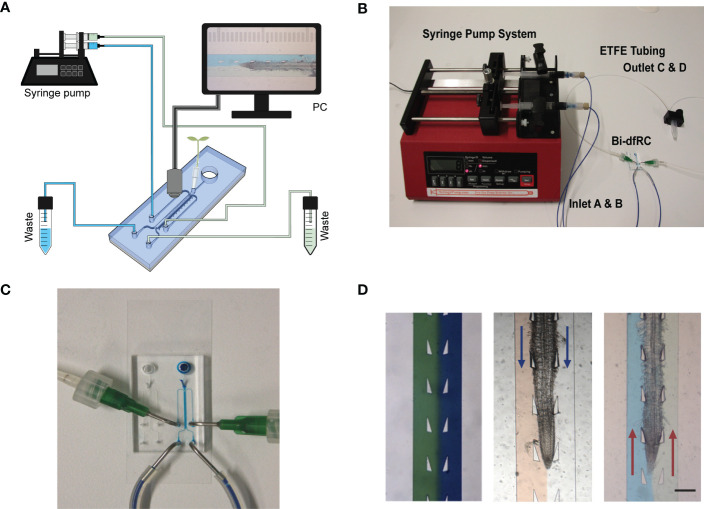
Bi-directional dual-flow-RootChip (Bi-dfRC) imaging set up. **(A)** Schematic diagram of the bi-dfRC set up with syringe pump system and tubing array for imaging. **(B)** Photograph of the syringe pump system, tubing and chip adapter used for the delivery of asymmetric test solutions into the bi-dfRC microchannels. **(C)** Close-up depicting the fluid flow into the observation chamber delivered by tubing network into the bi-dfRC in the absence of a root using blue dye (scale bar 5 mm). **(D)** *Arabidopsis* 9-day old G-CaMP3 root under asymmetric fluidic flow visualised using coloured dye in the absence and presence (flow direction indicated with blue arrows for differentiation zone and red arrows for tip) of a root (scale bar 350 μm).

While increasing functionality, adding additional inlets/outlets to a microfluidic device has the potential to increase the complexity for flow control (**Figure 2A**). This may lead to air bubble formation in the bi-dfRC microchannel during pressurised, controlled and active syringe pumping (**Figure 2B**). Backflow and leakage are a common cause of air bubble formation in microfluidic devices, a consequence of fluid flow resistance and limited flow rate control (Nakayama et al., 2006). This phenomenon is commonly caused by PDMS tearing at the inlet/outlet channels, varying circuit components or the defined size and length of circuit tubing (Xue et al., 2012). Air bubble formation in the bi-dfRC also has the potential to disrupt asymmetric profusion of two test solutions at the same time, thus leading to the mixing of these (Olanrewaju et al., 2018).

To prevent the aforementioned issues, and achieve optimal fluid-flow rate control, air bubble generation was actively minimised using the following optimisations. To prevent cracking of PDMS inlets and subsequent open-loop circulation/backflow and leakage, ‘flexible ends’ for the low compliance ETFE tubing networks were constructed. Short sections of flexible tubing (Masterflex Tygon, DO-06409-16; L= 1 cm) were used to connect the stiffer tubing to pre-made 1 mm × 1 cm metal tubes with a 90-degree bend (Dispensing Tips, Nordson), as shown in **Figure 2C**. To expel air bubbles and dry out microchannels during fabrication, each chip was degassed for two hours (Karlsson et al., 2013; Asghar et al., 2016). Prior to on-chip root sub-culture, microchannels were pre-wetted with control media *via* passive pumping. To expel any air bubbles retained in the microchannels after this, negative pressure was generated *via* active syringe pump back pumping into the syringes, thus further reducing the size of any air bubble (Liu & Li, 2015). Lastly, to avoid air bubble introduction upon media injection *via* the tubing array, microchannels harboring roots were pre-flushed with control media and inlets were primed with media droplets. In tandem, the tubing was primed with desired treatment by pre-pumping the media through the tubing array. Both droplets were touched together, creating a wet seal, avoiding bubble introduction.

Fine tuning flow rate control, air bubble formation and system set up presented a dual process to optimise steady laminar flow perfusion of test solutions. In order to observe successful asymmetric flow of simultaneous test solutions in the bi-dfRC OCs, coloured dyes were first pumped into the microchannel (**Figure 2D**). Flow rate control and tubing array maintain the delivery of asymmetric flow at the same constant speed of 20 µl/min for both sites. As observed in **Figure 2D**, solution(s) injected on each side of the root remain on the initial side without observed diffusion. Hence, two different stimulation solutions do not cross-mix when applied on different sides of the root. This highlighted the retention of co-flow in the bi-dfRC microchannels in the presence of an *Arabidopsis* root (**Figure 2D**).

### 3.4 NaCl and PEG induce an early calcium burst in the primary root

Calcium signals have been shown to be involved in early plant defence sensing, and response to salt and drought stress (Knight et al., 1997; Kiegle et al., 2000; Li et al., 2022). Prior studies tracked Ca^2+^ signals utilising various green fluorescent sensors including R-GECO1 (Stanley et al., 2018), YCNano-65 (Choi et al., 2014) and MatryoshCaMP6s (Ast et al., 2017). Based on refined spatio-temporal resolution and sensitivity, G-CaMP3 expressing *Arabidopsis* lines (Bi et al., 2021), with fast responsiveness towards Ca^2+^ oscillations were chosen to study the early Ca^2+^ response upon NaCl-induced salinity stress and PEG-induced drought stress. It should be noted that limitations exist with Ca^2+^ sensor technology, including Ca^2+^ quenching (Ca^2+^ sensor binding to Ca^2+^) and buffering the Ca^2+^ response (Granqvist et al., 2012). Additionally, it has been reported that some Ca^2+^ sensors are more sensitive towards physiological response mechanisms, such as G-CaMP6 reporting Ca^2+^ bursts from single action potentials (Cho et al., 2017). Moreover, variations of such factors may have an underlying effect on the observable physiological response of the root towards various stressors, limiting spatiotemporal resolution of the Ca^2+^ signal. As such, comparison of experimental observations using different sensors can be difficult at times due to the varying kinetics. Consequently, differences in observed Ca^2+^ spatio-temporal transmission within the literature is expected and will be further discussed in the sections below.

#### 3.4.1 The initial contact site with NaCl and PEG affect Ca^2+^ signal direction

Ca^2+^ signals primarily initiated in root tissue directly exposed to NaCl or PEG, then dispersed shoot or root ward depending on the first contact site of the stressor. The solution moves through the channel with a constant flow rate of 20 µL/min, due to the known dimensions of the channel and the root we can calculate that the solution needs ~1.76 sec to fill the 12 mm channel, when the root has grown 6 mm (50%) into the channel. Hence, the solution moves through the bi-dfRC with a speed of ~6.84*10^3^ µm/s. In contrast, the observed Ca^2+^ signal in the root induced by NaCl or PEG move at a speed of 4-14 µm/s, which is far slower than the solution. Therefore, it can be excluded that the flow of the solution in the channel of the chip is responsible for the movement of the Ca^2+^ signal within the root. NaCl-induced salinity stress and PEG-induced drought stress both reduce water absorption and obstruct water movement, inducing downstream osmotic and oxidative stress pathways (Ma et al., 2020). Here, we show differences in signal heterogeneity during local cellular and systemic Ca^2+^ signaling in roots responding to alternative types of osmotic stress inducing compounds.

**Figure 3:**
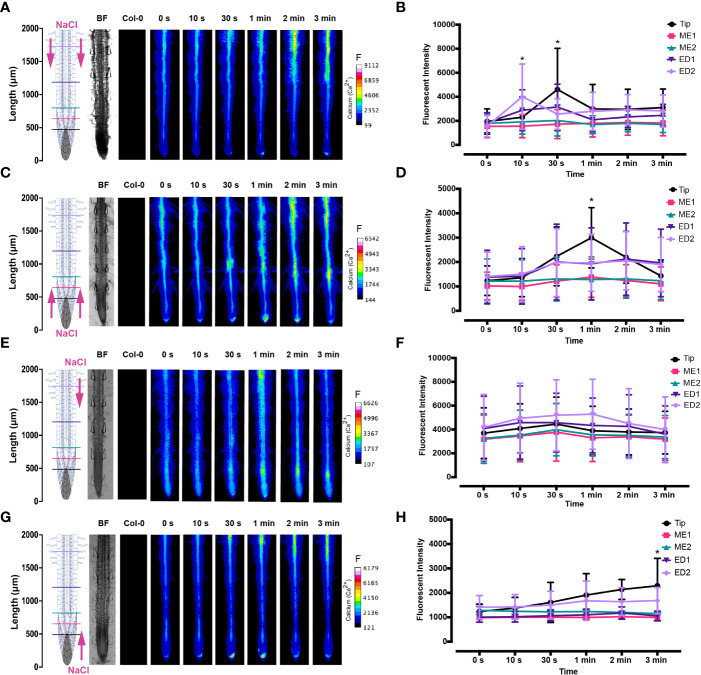
Fluorescence intensity of Ca^2+^ in *Arabidopsis* G-CaMP3 roots exposed to NaCl (100 mM). Fluorescence was observed for 180 s post NaCl treatment. **(A)** Heat map depicting Ca^2+^ release, corresponding to increase in G-CaMP3 fluorescence. Five linear sections used for fluorescence quantification upon targeted application of NaCl treatment through inlets A (left) & B (right) at the differentiation zone (DZ) are shown (n= 10). Colour change indicates an increase in Ca^2+^ fluorescence. Root schematic depicting treatment application, orientation (salt; NaCl and control; MS media) and linear sections (refer to key), bright field (BF) and control (wild type Col-0) roots displayed on the left. Scale; F= fluorescence intensity. **(B)** Line graph with two-way ANOVA multiple comparisons Tukey’s honestly significant difference (HSD) mean comparison test (P-value ≤ 0.05) depicting average fluorescence intensity (ADU; analogue digital units) of Ca^2+^ across five linear sections (Tip, ME1, ME2, ED1 & ED2) upon targeted exposure of salt treatment through inlets A & B at the DZ (n = 10). Asterisks (*) indicate statistical significance. **(C)** Heat map depicting Ca^2+^ release, and corresponding increase in G-CaMP3 fluorescence, upon salt treatment through inlets C (left) & D (right) at the tip (n= 10). **(D)** Line graph depicting average fluorescence intensity of Ca^2+^ across five linear sections following salt treatment through inlets C & D at the tip (n= 10). **(E)** Heat map depicting Ca^2+^ release, and corresponding increase in G-CaMP3 fluorescence, upon salt treatment through inlet B and control media through inlet A at the DZ (n= 10). **(F)** Line graph depicting average fluorescence intensity of Ca^2+^ across five linear sections following salt treatment through inlet B and control treatment through inlet A at the DZ (n= 10). **(G)** Heat map depicting Ca^2+^ release, and corresponding increase in G-CaMP3 fluorescence, upon salt treatment through inlet D and control treatment through inlet C at the tip (n= 5). **(H)** Line graph depicting average fluorescence intensity of Ca^2+^ across five linear sections upon salt treatment through inlet D and control through inlet C at the tip (n= 5).

**Figure 4:**
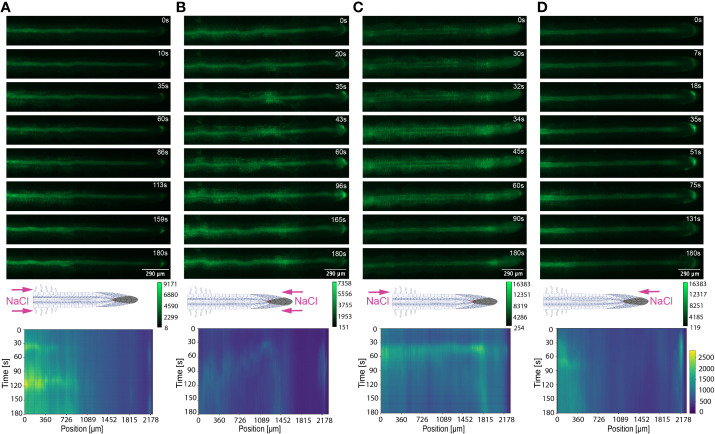
Key Ca^2+^ signal localisation in *Arabidopsis* roots exposed to 100 mM NaCl. Schematic diagrams depict treatment localisation and orientation at the root within the bi-dfRC with fluorescent intensity calibration bars. Kymographs depict the spatial fluorescense of GFP corresponding to Ca^2+^ in the root over time, in dependence of treatment orientation and localisation. GFP fluorescence in kymographs is color coded, ranging from dark blue to yellow (normalized for all samples). **(A)** Key Ca^2+^ localisation pattern upon NaCl treatment through inlets A & B of the bi-dfRC at the differentiation zone. **(B)** Key Ca^2+^ localisation pattern upon NaCl treatment through inlets C & D of the bi-dfRC at the tip. **(C)** Key Ca^2+^ localisation pattern upon NaCl treatment through inlet B (top) and control treatment through inlet A (base) of the bi-dfRC at the differentiation zone. **(D)** Key Ca^2+^ localisation pattern upon NaCl treatment through inlet D (top) and control treatment through inlet C (base) of the bi-dfRC at the tip.

**Figure 5:**
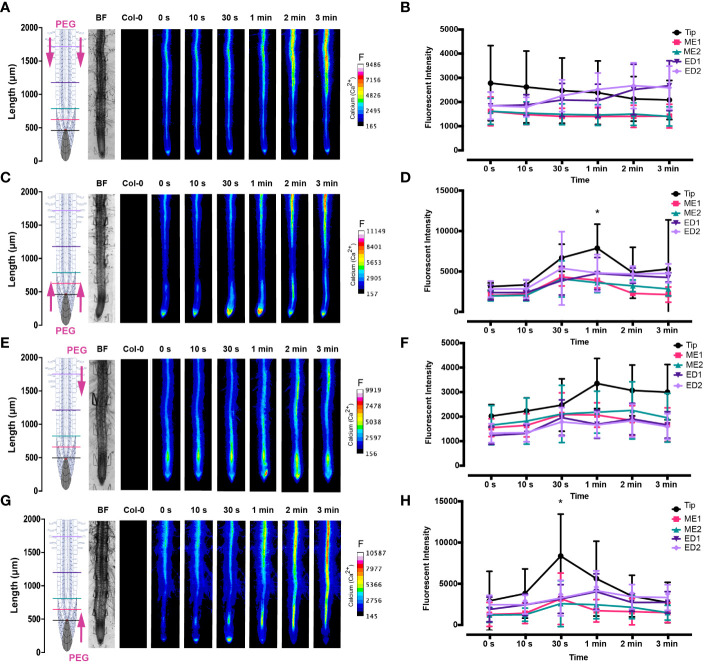
Fluorescence intensity of Ca^2+^ in *Arabidopsis* G-CaMP3 roots exposed to PEG (20%). Fluorescence was observed for 180 s post PEG treatment. **(A)** Heat map view depicting Ca^2+^ release, corresponding to increase in G-CaMP3 fluorescence. Five linear sections used for fluorescence quantification upon targeted application of PEG treatment through inlets A (left) & B (right) at the differentiation zone (DZ) are shown (n= 5). Colour change indicates an increase in Ca^2+^ fluorescence. Root schematic depicting treatment application, orientation (Polyethylene glycol; PEG and control; MS media) and linear sections (refer to colour key), bright field (BF) and control (wild type Col-0) roots displayed on the left. Scale; F= fluorescence intensity. **(B)** Line graph with two-way ANOVA multiple comparisons Tukey’s honestly significant difference (HSD) mean comparison test (P-value ≤ 0.05) depicting average fluorescence intensity (ADU; analogue digital units) of Ca^2+^ across 5 linear sections (Tip, ME1, ME2, ED1 & ED2) upon targeted exposure of PEG treatment through inlets A & B at the DZ (n = 5). Asterisks (*) indicate statistical significance. **(C)** Heat map depicting Ca^2+^ release, and corresponding increase in G-CaMP3 fluorescence, upon PEG treatment through inlets C (left) & D (right) at the tip (n= 5). **(D)** Line graph depicting average fluorescence intensity of Ca^2+^ across 5 linear sections following PEG treatment through inlets C & D at the tip (n= 5). **(E)** Heat map depicting Ca^2+^ release, and corresponding increase in G-CaMP3 fluorescence, upon PEG treatment through inlet B and control media through inlet A at the DZ (n= 5). **(F)** Line graph depicting average fluorescence intensity of Ca^2+^ across 5 linear sections following PEG treatment through inlet B and control treatment through inlet A at the DZ (n= 5). **(G)** Heat map depicting Ca^2+^ release, and corresponding increase in G-CaMP3 fluorescence, upon PEG treatment through inlet D and control treatment through inlet C at the tip (n= 5). **(H)** Line graph depicting average fluorescence intensity of Ca^2+^ across 5 linear sections upon PEG treatment through inlet D and control through inlet C at the tip (n= 5).

**Figure 6:**
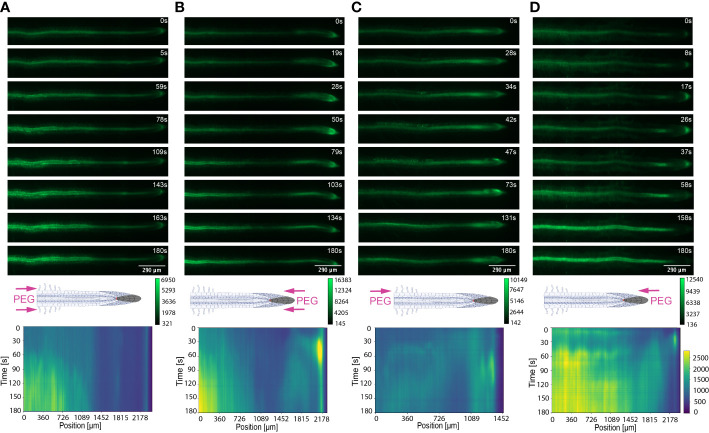
Key Ca^2+^ signal localisation in *Arabidopsis* roots exposed to 20% PEG. Schematic diagrams depict treatment localisation and orientation at the root within the bi-dfRC with fluorescent intensity calibration bars. Kymographs depict the spatial fluorescense of GFP corresponding to Ca^2+^ in the root over time, in dependence of treatment orientation and localisation. GFP fluorescence in kymographs is color coded, ranging from dark blue to yellow (normalized for all samples). **(A)** Key Ca^2+^ localisation pattern upon PEG treatment through inlets A & B of the bi-dfRC at the differentiation zone. **(B)** Key Ca^2+^ localisation pattern upon PEG treatment through inlets C & D of the bi-dfRC at the tip. **(C)** Key Ca^2+^ localisation pattern upon PEG treatment through inlet B (top) and control treatment through inlet A (base) of the bi-dfRC at the differentiation zone. **(D)** Key Ca^2+^ localisation pattern upon PEG treatment through inlet D (top) and control treatment through inlet C (base) of the bi-dfRC at the tip.

The spatio-temporal Ca^2+^ signals responding to environmental stress are shared between many organisms, including animals and plants (Clapham, 2007; Manishankar et al., 2018). In plant leaves and root tissues, increase of cytosolic Ca^2+^ has previously shown local osmotic stress applications (Liu et al., 2010; Choi et al., 2014; Ast et al., 2017; Huang et al., 2017; Stanley et al., 2018; Zhang et al., 2020). The signal has been documented as a ‘wave’ that initiated from site-specific NaCl stress at the lateral root (Choi et al., 2014). Specifically, the signal observed in Choi et al., 2014 initiated at the tip, emanated through cortical and endodermal cells of the lateral root, yet was not observed in epidermal cells. The Ca^2+^ wave then traversed through the primary root, splitting bi-directionally shoot and tip-ward, at a speed of 400 µm/s. The signal also systemically transmitted to above regions of the plant, moving through the hypocotyl (Choi et al., 2014) and leaves (Xiong et al., 2014). We show Ca^2+^ signal localisation and orientation in primary roots responding to a full or one-sided NaCl and PEG solution at the tip or differentiation zone. Based on former literature that observed Ca^2+^ signaling in response to NaCl treatment, 100 mM NaCl was chosen for the presented experiments (Choi et al., 2014; Stanley et al., 2018). Effects of 100 mM NaCl treatment on *Arabidopsis* has been well studied in the past, which allows integration of our insights into the existing knowledge (Yang et al., 2019; Cackett et al., 2022). PEG-6000 is known to induce drought stress *via* lowering plants water potential as a result of osmotic stress (Sevindik et al., 2022). Prior observations revealed a strong physiological drought inducing response at a concentration of 20% (Hellal et al., 2018; Huang et al., 2021). In complement, preliminary data detailed in Allan, 2021 showed 20% PEG induced the strongest Ca^2+^ response compared to lower fluorescence intensities observed at 0%, 5%, 10% and 40% concentrations. All G-CaMP3 and Col-0 control figures and videos are provided in the supplemental material (**Supplemental Figure 2**; **Supplemental Videos 9**, **10**). Ca^2+^ increased at epidermal cells of the differentiation zone upon first contact with the NaCl stressor applied at the shoot site. The propagating Ca^2+^ signal increased in intensity 1.5-fold and transmitted from the initial site, moving through the cortical and endodermal cells on both sides of the root, to the stele tissue. The signal also dispersed tip-ward through the epidermal, cortical and stele tissue, followed by a systemic increase of Ca^2+^ at the tip (P-value ≤ 0.02). The signal transferred longitudinally through the cortical and stele tissues, at a speed of 6.8 μm/s (SD = 0.9, n = 10) (**Figures 3A, B**, **4A**; **Supplemental Figures 3A, B**, **5**; **Supplemental Video 1**). The same Ca^2+^ signal was observed following PEG treatment at the differentiation zone, increasing in intensity 1.5-fold. Opposingly to the signal observed under salt, Ca^2+^ propagated from the endodermis/cortex into adjacent tissues, then traveled tip-ward at a faster speed of 11.6 µm/s (SD =1.6, n = 5) (**Figures 5A, B**, **6A**; **Supplemental Figures 4A, B**, **5**; **Supplemental Video 2**).

Conversely, following full NaCl treatment at the tip, Ca^2+^ upregulated at the columella cells, then increased in intensity 2.5-fold (P-value ≤ 0.05). This was followed by a Ca^2+^ wave that traveled shoot-ward throughout the stele tissue of the elongation zone to the differentiation zone at a speed of 5.9 µm/s (SD = 0.4, n = 10) (**Figures 3C, D**, **4B**; **Supplemental Figures 3C, D**, **5**; **Supplemental Video 3**). The same signal was observed following PEG treatment at the tip, whereby the signal increased 4-fold at the columella cells (p-value ≤ 0.0017). This signal was stronger in the endodermis and cortex, yet traveled shoot-ward at an increased speed of 10.5 µm/s (SD =1.6, n = 5) (**Figures 5C, D**, **6B**; **Supplemental Figures 4C, D**, **5**; **Supplemental Video 4**). Summarising, different root tissues are involved in local and systemic transmission of early Ca^2+^ signals in response to two different types of osmolyte. The resulting temporal signal patterns depended on the first contact site (differentiation zone or tip) and the inducing stressor. Early studies showed Ca^2+^ release, and corresponding increase in G-CaMP3 fluorescence, initiated in the root system upon immersion of entire 6 to 7-day old plantlets in NaCl (Knight et al., 1997; Kiegle et al., 2000). Patch clamp studies utilising protoplasts from maize root tips also showed cytosolic Ca^2+^ increased following PEG treatment (Liu et al., 2010). Later studies on the spatio-temporal NaCl induced-Ca^2+^ burst in lateral roots showed signal heterogenicity in the tissue and cell specificity (Choi et al., 2014; Ast et al., 2017). In roots expressing the ratiometric MatryoshCaMP6s transgenic detector, Ca^2+^ initially upregulated within defined cells of the root cap 4 s following NaCl application at the tip, then increased in intensity, while travelling through lateral root cap, epidermis and cortex within 39 s (Ast et al., 2017). Prior observations also showed a Ca^2+^ burst in different cells of *P. edulis* root tips, induced by submerging the planets in PEG for 10 minutes (Jing et al., 2019). Site specifically applying NaCl at the region where the lateral root protruded from the primary root revealed Ca^2+^ signaling originated at the localised cells of the cortical and endodermal cell layers (Choi et al., 2014). As discussed, this was followed by fast systemic signal propagation throughout the root expressing FRET based YCNano-65 sensor, depending on the conductance of Ca^2+^ through the ion channel protein Two Pore Channel 1 (TPC1) (Choi et al., 2014). A much slower Ca^2+^ signal was observed compared to Choi et al., 2014. This discrepancy may be due to the use of a bi-dfRC system combined with the G-CaMP3 sensor, compared to an agarose gel-based system incorporated with the FRET based YCNano-65 sensor (Choi et al., 2014). Moreover, YCNano-65 has been shown to have a dissociation constant (K_d_) of 64.8 nM (Horikawa et al., 2010), compared to 660 nM in G-CaMP3 (Tian et al., 2009), which may lead to a faster documented signal, but also different physiological effects on the sensor line overall.

Interestingly, the initial Ca^2+^ release, and corresponding increase in G-CaMP3 fluorescence, and longitudinal dispersion of Ca^2+^ following primary root tip exposure to NaCl and PEG in G-CaMP3 transgenic roots reported here was slower, yet comparable to observations in Ast et al., 2017, utilising ratiometric MatryoshCaMP6s expressing roots. Additionally, the systemic transmission of Ca^2+^ through varying tissues following initial Ca^2+^ release, and corresponding increase in G-CaMP3 fluorescence, at both the differentiation zone or tip treatment sites showed a similar tissue-specific dispersion pattern.

#### 3.4.2 Different transverse and longitudinal tissue specific Ca^2+^ signals arise following one-sided NaCl and PEG treatment

The NaCl and PEG-induced Ca^2+^ signals initiated on the treated side of the root. Observations showed varying cell types and tissues were involved in the spatio-temporal dispersion of Ca^2+^, in dependence on the initial stress site. A one-sided NaCl or PEG treatment at the differentiation zone always led to a transversely moving signal, from the contact to the non-contact side. The signal then moved longitudinally to the tip. Upon NaCl treatment at the differentiation zone, the Ca^2+^ burst emanated from epidermal cells spanning the elongation and differentiation zone on the treatment side of the root, in addition to the root tip. The signal increased 1.5-fold in intensity and transversely moved from the treated to untreated side of the root at a speed of 9.4 μm/s. A secondary Ca^2+^ wave was observed, which moved longitudinally to the root tip through stele tissue at a speed of 5.2 μm/s (SD = 0.57, n= 10) (**Figures 3E, F**, **4C**; **Supplemental Figures 3E, F**, **5**; **Supplemental Video 5**). PEG induced the same transverse signal response yet this was faster, less intense and did not transmit to the untreated side of the root. Additionally, the secondary longitudinal Ca^2+^ signal transmitted not only tip-ward, but bi-directionally, root and shoot ward at a speed of 11 μm/s (SD = 1.4, n= 5) through cortical and stele tissue. (**Figures 5E, F**, **6C**; **Supplemental Figures 4E, F**, **5**; **Supplemental Video 6**). Prior observations show the NaCl-induced Ca^2+^ signal traveled transversely at a comparable rate 14.1 μm/s (Stanley et al., 2018). This traversing Ca^2+^ wave is only observed following one-side osmolyte treatment at the differentiation zone and not the tip. This suggests that the traversing Ca^2+^ signal observed is due to the Ca^2+^ signal itself, rather than the diffusion of NaCl or PEG into the root.

Excitingly, for the first time we show a one-side NaCl and PEG treatment from the tip. The Ca^2+^ burst localised on the right side of the columella cells, which then increased in intensity on the right side of the tip by 2.5-fold. Next, the Ca^2+^ signal dispersed shoot-ward through stele tissue and cortical tissue of the differentiation zone, at a speed of 4.2 μm/s (SD = 0.51, n= 5) (**Figures 3G, H**, **4D**; **Supplemental Figures 3G, H**, **5**; **Supplemental Video 7**). PEG induced a similar signal response (P-value ≤ 0.0441), however traveled faster at a speed of 14.2 µm/s (SD = 1.9, n= 5), and reaching a 0.5-fold higher intensity (**Figures 5G, H**, **6D**; **Supplemental Figures 3G, H**, **5**; **Supplemental Video 8**).

The associated kymographs show that Ca^2+^ signals can orient in different directions based on the initial NaCl or PEG treatment site. Moreover, both PEG and NaCl induced a strong Ca^2+^ burst at the tip if the stress is applied locally. However, PEG induced a stronger signal throughout the differentiation zone in all treatments (**Figures 4A, B**). Plant root responses to environmental stress are highly sensitive and differ at the cellular, tissue, and organ level (Duan et al., 2015). Moreover, site specific localisation of osmotic stress to varying tissues at the primary root suggests plants are well adapted to sense environmental stress directly in affected tissues. Building on early observations, we show the initial Ca^2+^ signal in the primary root is not only site-specific to cells exposed to locally applied osmotic stressors, but also systemically transmits away from initially stimulated cells, at the tip and differentiation zone. Additionally, the Ca^2+^ wave passed through different root tissues and cells depending on the initial site of Ca^2+^ release, and corresponding increase in G-CaMP3 fluorescence. Osmotic stress inhibits plant root growth and development *via* redistribution of the stress hormone auxin from the quiescent center (QC) and root cap to the epidermal and cortical cells of the elongation zone, causing the root to bend away from high salt concentrations (Smolko et al., 2021). Additionally, when cells are exposed to auxin, membrane-bound proton pumps export H^+^, decreasing apoplastic pH and ultimately leading to cell wall loosening – known as the acid growth hypothesis (Arsuffi & Braybrook, 2018). Prior studies showed that Ca^2+^ signaling participated in root growth and stress sensing *via* modulating auxin responses to abiotic stress (Shih et al., 2015; Dindas et al., 2018; Leitão et al., 2019).

The different Ca^2+^ signal patterns responding to NaCl and PEG in varying orientations and localisations observed here may be intrinsically linked to auxin movement and root growth under osmotic stress. Further studies will be needed to clearly link the exact signaling mechanisms.

We also observed varying rates of longitudinal Ca^2+^ transmission depending on the localisation and orientation of NaCl or PEG treatment. Prior observations showed that signal cross-talk exists between extracellular reactive oxygen species (ROS) hydrogen peroxide (H_2_O_2_), and intracellular Ca^2+^ for systemic propagation of the signal between varying cells and tissue (Gilroy et al., 2014; Stanley et al., 2018). Longitudinal signal transmission rates can vary based on the number of cell boundaries present within varying root tissues (Gilroy et al., 2014). Moreover, the different longitudinal tissue specific signals observed between full and one-sided NaCl or PEG treatment may generate varying signal speeds. Such findings suggest that differences in cell types and tissues may regulate how the Ca^2+^ signals are sensed, transmitted over space and time and decoded into a response. This includes the existence of 1085 distinct proteins associated with Ca^2+^ binding and/or calcium ion sensor activity (Allan et al., 2022a). Employment of such cell or stimulus specific decoders and responders in roots may impact the transmission pattern of Ca^2+^ transient signatures across varying tissues for long distance signaling, in dependence of the exact localisation of an external stress in the soil. Overall, the presented research focused on optimising a bi-dfRC microfluidic device that allowed precise tracking of root Ca^2+^ signal directionality in response to stress solution in varying orientations and localisations. Summarising, we reported novel insights into the traverse and longitudinal directionality of osmolyte induced-Ca^2+^ signals. These signals were transmitted root or shoot-ward in depends of on the first contact site with the stress solution. Results presented here suggest that plants may use different sensing machinery in response to abiotic stress conditions. However, more research is required to confirm that the varying signal responses may fine tune adaptation processes.

## 4 Conclusion

Advances in bi-dfRC technology have revealed fascinating Ca^2+^ signal patterns in response to osmotic stress in varying localisations and orientations in the primary root. We show both NaCl and PEG treatments induced a Ca^2+^ signal that initially upregulated at the cells in first contact with the stressor. Following osmolyte treatment at the root tip, the Ca^2+^ signal initiated at the columella cells. Whereas, osmolyte treatment at the shoot site resulted primarily in cytosolic Ca^2+^ increase in the epidermal and cortical tissues of the differentiation zone. The following systemic transmission of Ca^2+^ is always oriented away from the initial contact site, and propagated faster in PEG than NaCl treated roots. Interestingly the signal moved longitudinally through different cell types depending on the localisation and orientation of the stress. Additionally, a one-sided NaCl or PEG treatment at the shoot site induced a Ca^2+^ signal that primarily traveled transversely through the root. The signal patterns observed here are complex, given that the root is a cylindrical 3D object compared to leaves, which can be simplified as a cuboid object. Hence, present results are limited by our 2D observations, due to the given speed limit of imaging processes to date.

Many Ca^2+^ sensors are now implemented in research, so it is important to note that their kinetics widely vary (Mertes et al., 2022). The G-CaMP3 sensor uitilised here harbours increased sensitivity and binding affinity compared to prior G-CaMP family sensors (Tian et al., 2009). However, more recently engineered Ca^2+^ sensors, including FRET-based MatryoshCaMP6s, exhibit superior fluorescence, dynamic range and sensitivity (Ast et al., 2017). Consequently, limitations of spatiotemporal resolution still exist using the G-CaMP3 sensor, as the signal speed and intensity observed may vary in prior and future studies, depending on the system and fluorescence indicator chosen. Moving forward, there is a need to be thoughtful about the side effects fluorescent sensors may have on plant physiological stress responses towards varying environmental stressors. The presented bi-dfRC application is not restricted to NaCl and PEG-induced Ca^2+^ stress signaling. We propose the asymmetric laminar flow capabilities of the bi-dfRC will present a broad application basis to investigate defence signaling in response to abiotic osmolytes and biotic peptides, and the combinatory effects of both. Future applications will also provide insight into possible paralleled signaling between Ca^2+^ and the ROS H_2_O_2_, in addition to downstream signaling including nitric oxide (NO) and lipids, while observing and tracking RNA and protein movement within root cells. We believe that this technology will position us better for future studies that will potentially lead to novel insights into mechanisms of molecular adaptations that underlie improved tolerance and survival of crop plants to challenges imposed by climate and pathogens.

## Data availability statement

The raw data supporting the conclusions of this article will be made available by the authors, without undue reservation.

## Author contributions

C-NM and VN supervised the experiments. CA performed and designed the experiments and analysed the data. R.H. wrote the script for Ca^2+^ quantification and Kymogram analysis. VN designed and prepared the wafer for the chips. AT provided technical assistance to CA. C-NM and VN conceived the project. CA wrote the article with contributions of all the authors. C-NM and VN supervised and completed the writing. C-NM agrees to serve as the author responsible for contact and ensures communication. All authors contributed to the article and approved the submitted version.

## Supplementary material

Supplementary Video 1 An *Arabidopsis* G-CaMP3 root exposed to 100 mM NaCl at the differentiation zone via inlets A & B of the bi-dfRC. At time interval 20 s, mature cells are primarily stimulated (GFP). The playback rate is 30 fps. Scale bar, 290 μm.

Supplementary Video 2 An *Arabidopsis* G-CaMP3 root exposed to 20% PEG at the differentiation zone via inlets A & B of the bi-dfRC. At time interval 30 s, mature cells are primarily stimulated (GFP). The playback rate is 30 fps. Scale bar, 290 μm.

Supplementary Video 3 An *Arabidopsis* G-CaMP3 root exposed to 100 mM NaCl at the tip via inlets C & D of the bi-dfRC. At time interval 10 s, tip cells are primarily stimulated (GFP). The playback rate is 30 fps. Scale bar, 290 μm.

Supplementary Video 4 An *Arabidopsis* G-CaMP3 root exposed to 20% PEG at the tip *via* inlets C & D of the bi-dfRC. At time interval 10 s, tip cells are primarily stimulated (GFP). The playback rate is 30 fps. Scale bar, 290 μm.

Supplementary Video 5 An *Arabidopsis* G-CaMP3 root exposed to 100 mM NaCl through inlet B (top) and control through inlet A (base) of the bi-dfRC at the differentiation zone. At time interval 25 s, mature cells are primarily stimulated (GFP). The playback rate is 30 fps. Scale bar, 290 μm.

Supplementary Video 6 An *Arabidopsis* G-CaMP3 root exposed to 20% PEG through inlet A (base) and control through inlet B (top) of the bi-dfRC at the differentiation zone. At time interval 30 s, mature cells are primarily stimulated (GFP). The playback rate is 30 fps. Scale bar, 290 μm.

Supplementary Video 7 An *Arabidopsis* G-CaMP3 root exposed to 100 mM NaCl through inlet D (top) and control through inlet C (base) of the bi-dfRC at the tip. At time interval 10 s, tip cells are primarily stimulated (GFP). The playback rate is 30 fps. Scale bar, 290 μm.

Supplementary Video 8 An *Arabidopsis* G-CaMP3 root exposed to 20% PEG through inlet D (top) and control through inlet C (base) of the bi-dfRC at the tip. At time interval 10 s, tip cells are primarily stimulated (GFP). The playback rate is 30 fps. Scale bar, 290 μm.

Supplementary Video 9 An *Arabidopsis* G-CaMP3 root exposed to control ½ MS/0.31 mM MES media through inlet A & B of the bi-dfRC at the differentiation zone. No Ca^2+^ burst is observed. The playback rate is 30 fps. Scale bar, 290 μm.

Supplementary Video 10 An *Arabidopsis* G-CaMP3 root exposed to control ½ MS/0.31 mM MES media through inlet C & D of the bi-dfRC at the differentiation zone. No Ca^2+^ burst is observed. The playback rate is 30 fps. Scale bar, 290 μm.
