# Supplementary material for: A dual-flow RootChip enables quantification of bi-directional calcium signaling in primary roots
Source: Front Plant Sci. 2023 Jan 10;13:1040117. doi: 10.3389/fpls.2022.1040117 (PMC9871814; doi:10.3389/fpls.2022.1040117)
Supplement: Supplementary file 1 [file DataSheet_1.pdf]

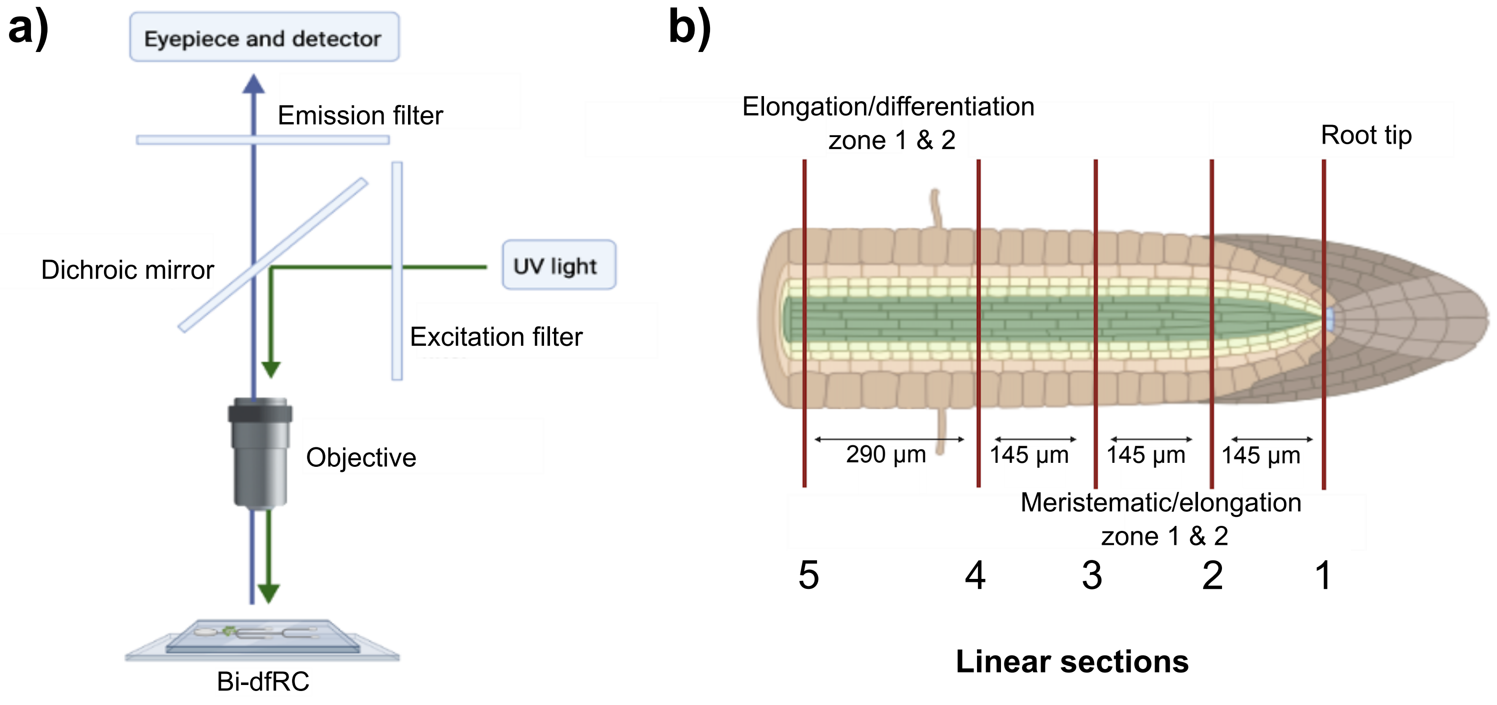


*Supplemental Figure 1. (a) Schematic diagram depicting the optical path of an epifluorescence microscope. (b) Schematic diagram depicting five linear sections used for data analysis designed to capture* $Ca^{2+}$ *fluorescent signal across three main zones of the* Arabidopsis *root; root tip (columella), meristematic/ elongation zone and elongation/differentiation zone.*
