# Supplementary material for: A dual-flow RootChip enables quantification of bi-directional calcium signaling in primary roots
Source: Front Plant Sci. 2023 Jan 10;13:1040117. doi: 10.3389/fpls.2022.1040117 (PMC9871814; doi:10.3389/fpls.2022.1040117)
Supplement: Supplementary file 2 [file DataSheet_2.pdf]

a)

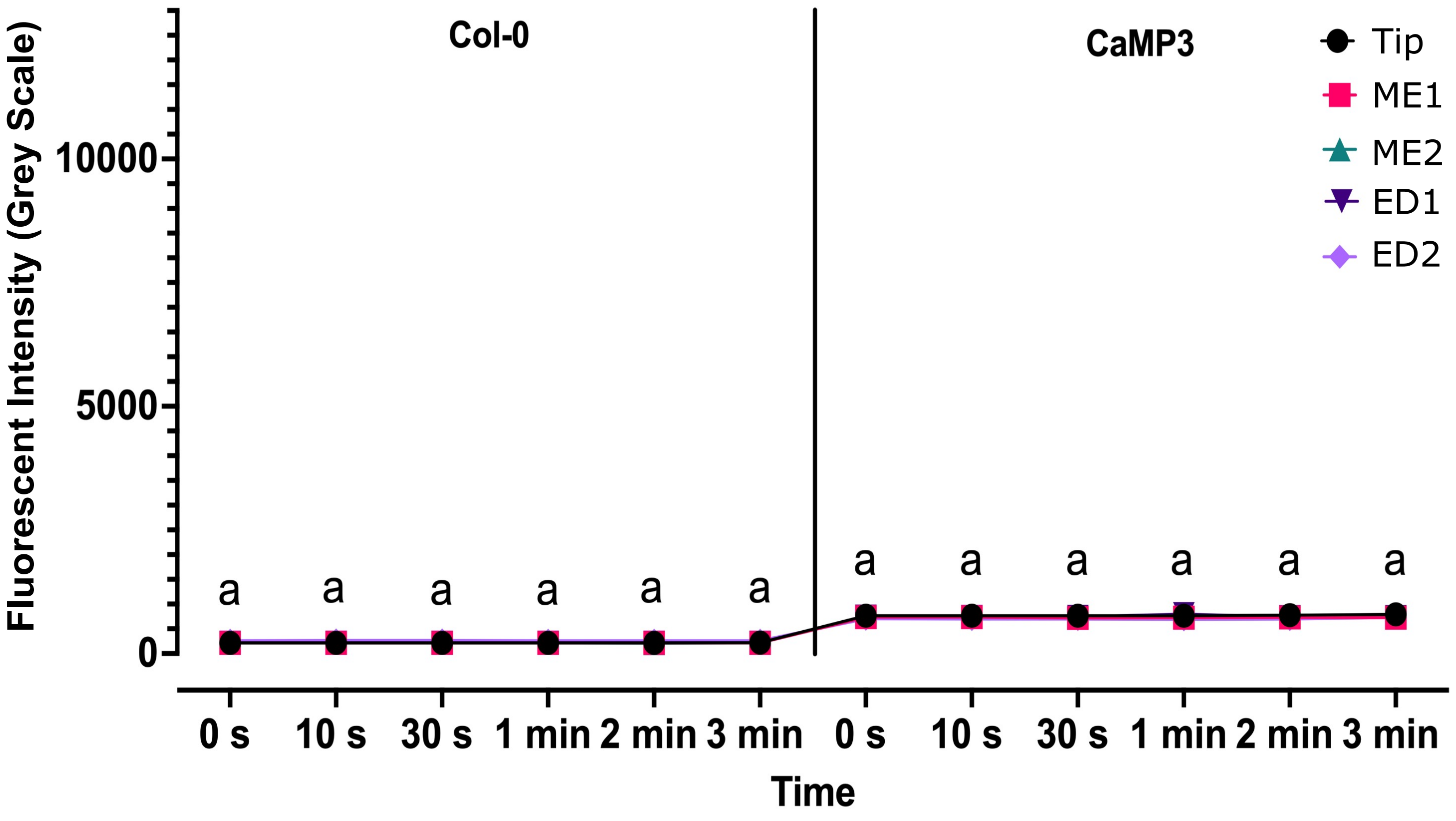

b)

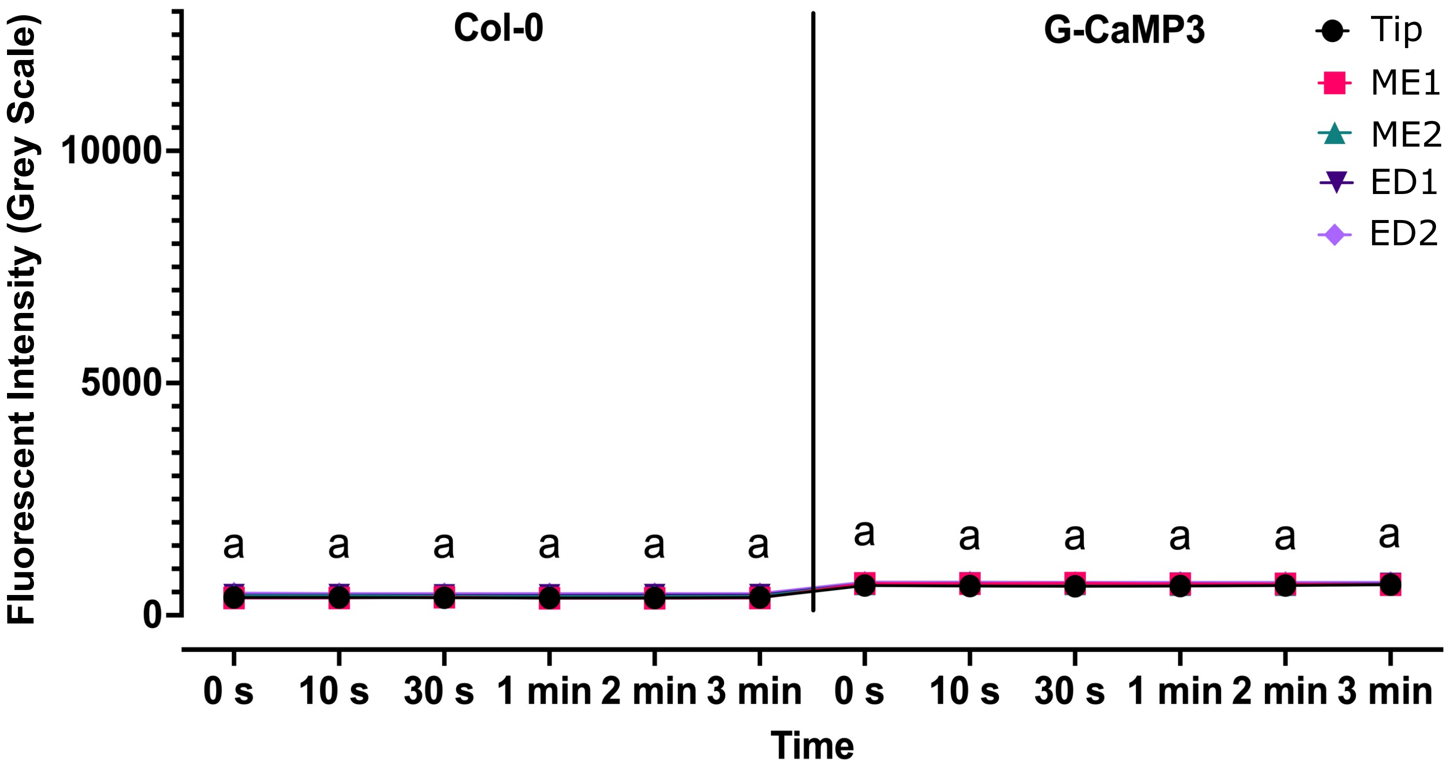

Supplemental Figure 2. Real-time fluorescence intensity tracking of Ca^{2+} in Arabidopsis *G-CaMP3 and Col-0 roots exposed to ½ MS/0.31 mM MES control media over 180 seconds. (a) Line graph with two-way ANOVA Tukey's honestly significant difference (HSD) mean comparison test (P-value ≤ 0.05) depicting fluorescence intensity (ADU; analogue digital units) of Ca^{2+} across five linear sections (Tip, ME1, ME2, ED1 & ED2) upon targeted exposure of full control treatment at the differentiation zone (n= 5) and, (b) full control treatment at the tip (n= 5).*
