# Supplementary material for: A dual-flow RootChip enables quantification of bi-directional calcium signaling in primary roots
Source: Front Plant Sci. 2023 Jan 10;13:1040117. doi: 10.3389/fpls.2022.1040117 (PMC9871814; doi:10.3389/fpls.2022.1040117)
Supplement: Supplementary file 4 [file DataSheet_4.pdf]

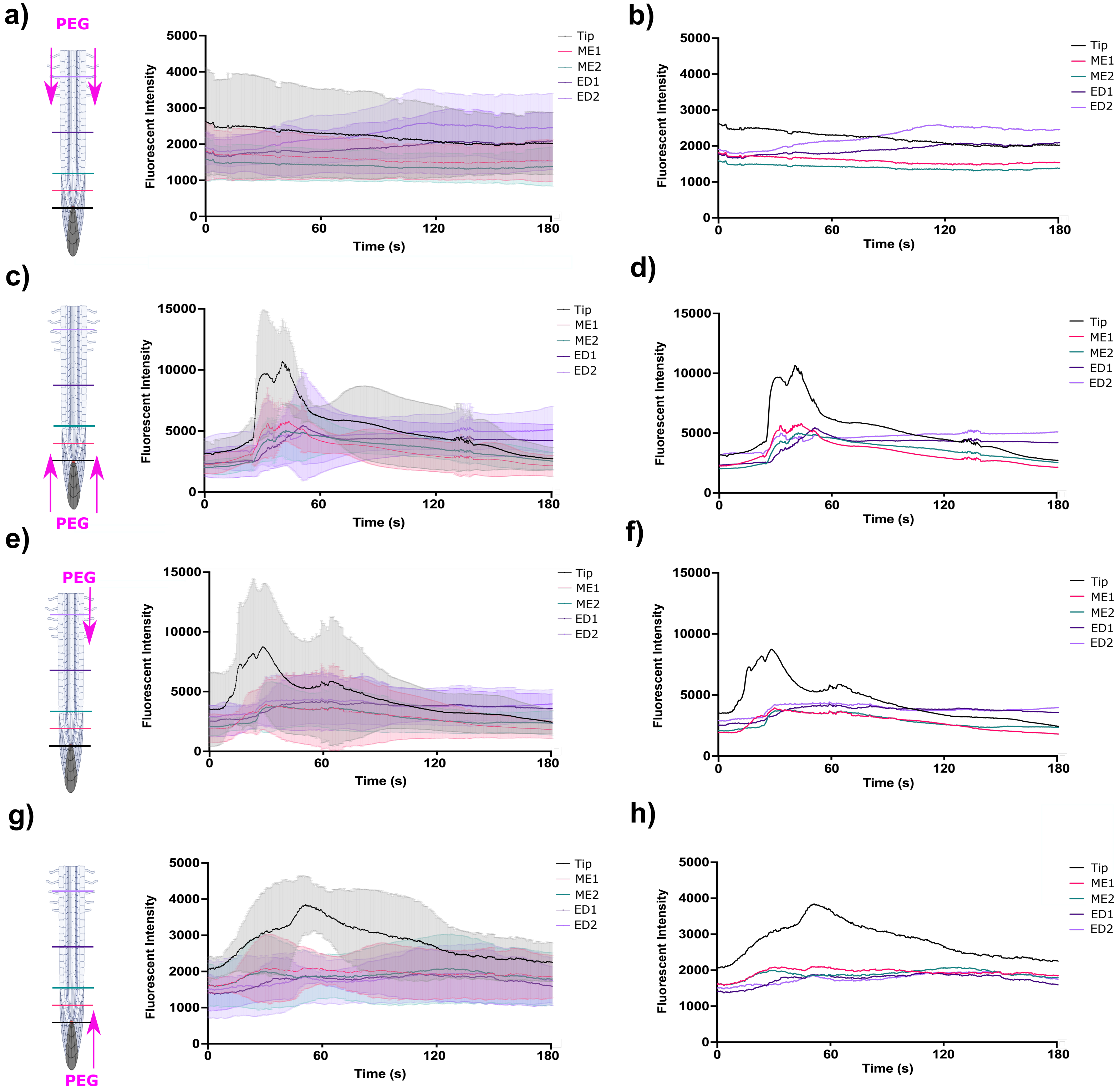

Supplemental Figure 4. Ca^{2+} release and corresponding increase in G-CaMP3 fluorescence, in Arabidopsis *primary roots was followed for 180 s post PEG (20%) treatment. Root schematics depict treatment application, orientation (Polyethylene glycol; PEG) and linear sections (refer to colour key). (a) Line graph depicting average fluorescence intensity (ADU; analogue digital units) of Ca^{2+} across five linear sections (Tip, ME1, ME2, ED1 & ED2) upon targeted exposure of PEG treatment through inlets A & B at the DZ (n = 5). Standard deviation included. (b) Line graph depicting mean fluorescence intensity (ADU; analogue digital units) of Ca^{2+} across five linear sections upon targeted exposure of PEG treatment through inlets A & B at the DZ (n= 5). (d) Line graph depicting average fluorescence intensity of Ca^{2+} across five linear sections following PEG treatment through inlets C & D at the tip (n= 5). Standard deviation included. (e) Line graph depicting mean fluorescence intensity of Ca^{2+} across five linear sections following PEG treatment through inlets C & D at the tip (n= 5). (e) Line graph depicting average fluorescence intensity of Ca^{2+} across 5 linear sections following PEG treatment through inlet B and control treatment through inlet A at the DZ (n= 5). Standard deviation included. (f) Line graph depicting mean fluorescence intensity of Ca^{2+} across five linear sections following PEG treatment through inlet B and control treatment through inlet A at the DZ (n= 5). (g) Line graph depicting average fluorescence intensity of Ca^{2+} across five linear sections upon PEG treatment through inlet D and control through inlet C at the tip (n= 5). Standard deviation included. (h) Line graph depicting mean fluorescence intensity of Ca^{2+} across five linear sections upon PEG treatment through inlet D and control through inlet C at the tip (n= 5).*
