# Supplementary material for: A dual-flow RootChip enables quantification of bi-directional calcium signaling in primary roots
Source: Front Plant Sci. 2023 Jan 10;13:1040117. doi: 10.3389/fpls.2022.1040117 (PMC9871814; doi:10.3389/fpls.2022.1040117)
Supplement: Supplementary file 5 [file DataSheet_5.pdf]

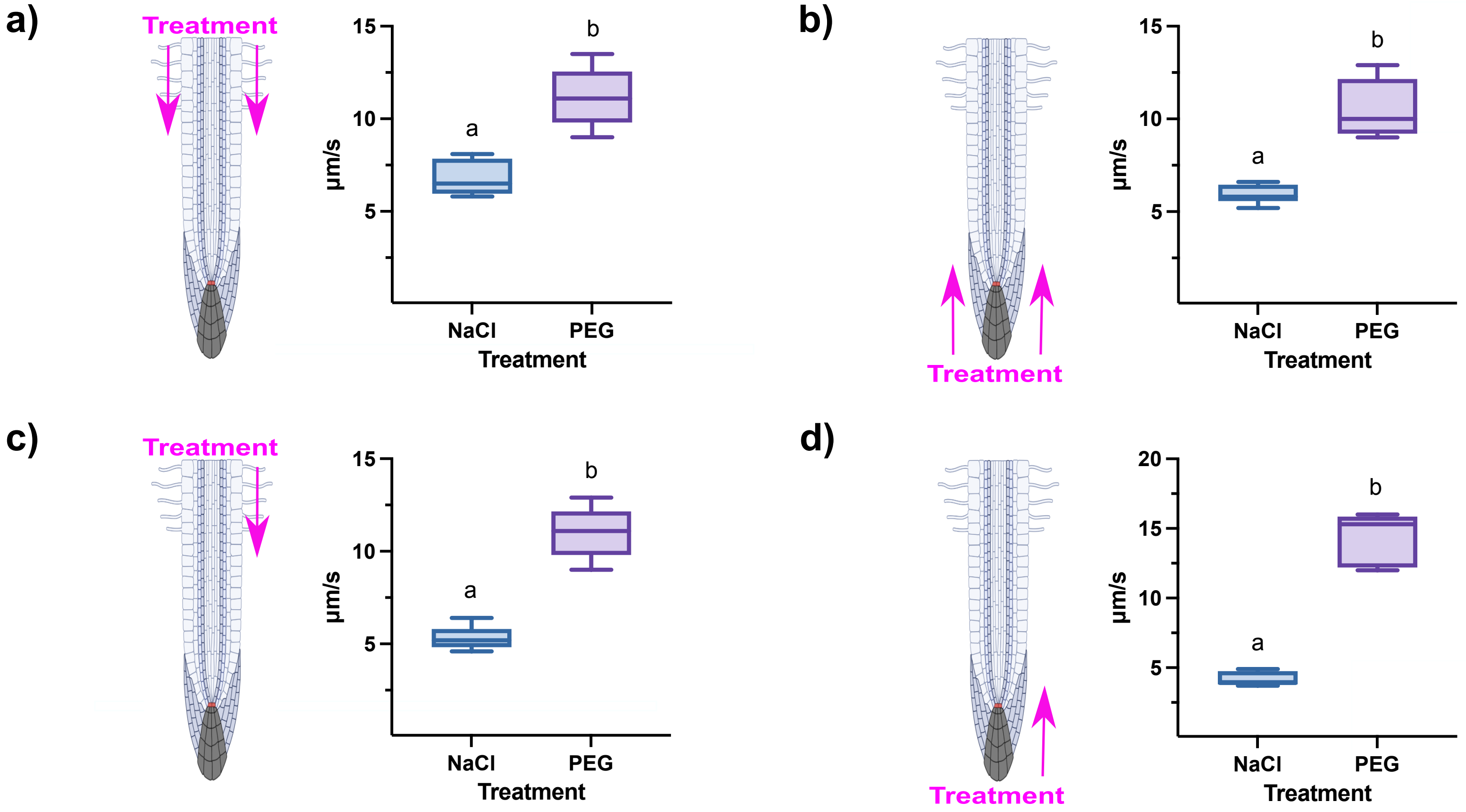

Supplemental Figure 5. Comparison of the Ca^{2+} signal transmission speed between full, one-sided NaCl and PEG treated samples at the differentiation zone and tip. Schematic diagrams of a root depicting treatment orientations and localisations. Box graphs with unpaired t test Welch's correction two-tailed test (P-value ≤ 0.01). (a) Longitudinal Ca^{2+} signal speed (µm/s) following full NaCl and PEG treatment at the differentiation zone, (b) full NaCl and PEG treatment at the tip, (c) one-sided NaCl and PEG treatment at the differentiation zone, (d) and one-sided NaCl and PEG treatment at the tip.
